# Two-photon directed evolution of green fluorescent proteins

**DOI:** 10.1038/srep11968

**Published:** 2015-07-06

**Authors:** Caleb R. Stoltzfus, Lauren M. Barnett, Mikhail Drobizhev, Geoffrey Wicks, Alexander Mikhaylov, Thomas E. Hughes, Aleksander Rebane

**Affiliations:** 1 Physics Department, Montana State University, Bozeman MT. 59717; 2 Cell Biology Neuroscience Department, Montana State University, Bozeman MT. 59717; 3 National Institute of Chemical Physics and Biophysics, Tallinn, Estonia, 12618.

## Abstract

Directed evolution has been used extensively to improve the properties of a variety of fluorescent proteins (FPs). Evolutionary strategies, however, have not yet been used to improve the two-photon absorption (2PA) properties of a fluorescent protein, properties that are important for two-photon imaging in living tissues, including the brain. Here we demonstrate a technique for quantitatively screening the two-photon excited fluorescence (2PEF) efficiency and 2PA cross section of tens of thousands of mutant FPs expressed in *E. coli* colonies. We use this procedure to move EGFP through three rounds of two-photon directed evolution leading to new variants showing up to a 50% enhancement in peak 2PA cross section and brightness within the near-IR tissue transparency wavelength range.

EGFP is recognized as the fluorescent probe of choice in most demanding two-photon imaging experiments because of its superior photostability and high fluorescence quantum yield^1^,^2^,^3^,^4^,^5^,^6^,^7^,^8^,^9^,^10^. However, the maximum 2PEF efficiency of EGFP, in particular its peak 2PA cross section of σ_2PA_ (900 nm) = 40 GM (1 GM = 10^−50^  cm^4^ s photon^−1^)^11^, lags behind the best values reported for some other types of FPs, notably the red FPs^11^, leaving ample room for improvement. Here we present a method of improving σ_2PA_ of green FPs using two-photon directed evolution. Attempts to increase the 2PEF efficiency by introducing smart point mutations into the EGFP framework have been hampered by the complex and mostly unknown relationship between σ_2PA_ and the protein structure^12^. Directed evolution offers an alternative route but requires fast, yet sufficiently accurate, screening of the two-photon properties of a large number of FP mutants^13^,^14^,^15^,^16^. Until now, this has proven to be an exceedingly challenging task. Even though one-photon excited fluorescence (1PEF) and 2PEF share some fluorescence characteristics, such as the emission wavelength and the emission yield, the 2PEF brightness is proportional to the value of σ_2PA_, which cannot be deduced from one-photon properties alone, making two-photon screening imperative. We address this issue by constructing a femtosecond 2PEF imaging setup that quantifies both the 1PEF and the 2PEF efficiency of tens of thousands of mutant FPs expressed in *E. coli* colonies. This allows us to pick mutants showing promising increases of σ_2PA_, which is an important property of FPs used in two-photon imaging of living tissues^9^,^15^,^17^,^18^,^19^,^20^.

The small σ_2PA_ values of FPs imply that large photon flux is needed to achieve a practical two-photon excitation rate. In a typical 2PEF microscope setup a minimum peak photon flux of ~10^28^ photons cm^−2^ s^−1^ is obtained by using ~100 MHz repetition rate, ~100 fs duration femtosecond oscillator laser pulses focused to a nearly diffraction-limited spot, which is then raster-scanned over a sample area of ~1 mm^2^ or less^18^,^21^,^22^,^23^. However, two-photon directed evolution experiments would require measuring the 2PEF from a much larger sample area, typically a standard (9 cm diameter) Petri plate, which is hardly practical, especially considering a maximum reasonable time frame of ~1 h per plate (see Supplementary Information for detailed evaluation of the technical issues involved). To accomplish this large area 2PEF imaging we therefore use a 1 kHz pulse repetition rate, 150 fs pulse duration regenerative amplifier beam shaped into a 2 × 20 mm^2^ stripe with an intensity of ~10^10^ J cm^−2^ s^−1^ on the sample, as shown in Fig. 1. We can scan this stripe over an entire Petri plate area within about 20 min, while the fluorescence image of the entire plate is captured by a CCD camera (see Methods and Supplementary Information for details on laser setup and imaging methods).

In the 2PEF image, the *E. coli* colonies appear as a collection of distinct small bright areas or spots, where each spot corresponds to the fluorescence from a particular mutant FP (see Supplementary Fig. 1). The 2PEF intensity integrated over the spot area is proportional to the two-photon brightness of the corresponding FP (defined as the product of σ_2PA_ and the fluorescence yield, see Supplementary Information). The integrated fluorescence also scales linearly with the total number of the mature FP chromophores in each colony, where the last parameter varies broadly from one colony to another depending on many secondary factors such as the bacteria replication rate, FP production, folding efficiency, protein maturation rate etc^13^,^24^, and is thus notoriously difficult to determine. Fortunately, we can take advantage of the fact that the integrated 1PEF signal has the same linear dependence on the FP chromophore concentration as the 2PEF signal. By measuring both the 2PEF and 1PEF from each colony and by evaluating the ratio between the integrated 2PEF and 1PEF values, we can effectively minimize the uncertainly due to varying expression and maturation rates, which allows us to evaluate the relative two-photon brightness of FP mutants on a whole plate in a reasonable amount of time. Furthermore, by calibrating the fluorescence of the mutated FPs with respect to a reference sample containing only non-mutated EGFP colonies, we can quantitatively compare the two-photon efficiency of a whole library of mutants, usually expressed on tens of different plates and measured at different times, to the non-mutated parent FP. Bringing all the above together, the screening parameter that we use to identify the most promising mutants may be expressed as the ratio between the normalized integrated 2PEF and 1PEF signals of the n^th^ mutant given by:


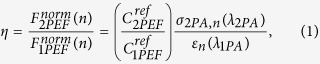


where σ_2PA,n_(λ_2PA_) is the two-photon absorption cross section of the n^th^ mutant at the excitation wavelength, λ_2PA_, ε_*n*_(λ_*1PA*_) is the molar extinction coefficient of the mutant at the one-photon excitation wavelength, λ_1PA_, and 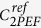 and 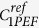 are, respectively, proportional to the average 2PEF and the average 1PEF signals of the reference sample (see Supplementary Information for details about the reference correction and this calculation). The quantity enclosed in the large brackets is constant for all samples in a particular library, which means that the screening parameter given by equation (1) turns out to be simply a constant times the ratio between the 2PA and 1PA cross section values at the respective excitation wavelengths.

The screening parameter given by equation (1) is not unique, in the sense that it depends on the choice of the excitation wavelengths λ_2PA_ and λ_1PA_. In principle, the wavelengths can be selected to guide the evolution in different directions e.g. shifting and/or maximizing the peak two-photon wavelength. In our current experiment, however, we are restricted to λ_2PA_ = 790 nm by our laser system, which is ~100 nm below the 2PA peak of EGFP. We chose the one-photon wavelength to also be below the 1PA peak, at λ_1PA_ = 450 nm, in order to maximize the effect that mutations can have on *η*, thus augmenting the efficacy of the screening procedure. This circumstance has consequences regarding the final evolution outcome, as will be described below.

Figure 2 summarizes the screening data from three consecutive cycles of evolution starting with EGFP, where the mutant libraries were obtained by a combination of error prone PCR and gene shuffling, and were expressed in *E. coli* colonies grown on ampicillin charcoal black agar Petri plates (see Supplementary Information). About 15,000 individual fluorescent colonies were screened in the three rounds of mutation, as summarized in Fig. 2. In the scatter graphs (left panel) each colony is plotted in the integrated and normalized coordinates of the 1PEF (horizontal axis) and 2PEF (vertical axis) signals. The red dots represent the mutagenized colonies, whereas the black dots correspond to the non-mutagenized reference colonies. The dashed diagonal line represents idealized non-mutagenized EGFP (*η*  =  1). In the right panel the same data is arranged in the form of a histogram, which shows the frequency of a particular 2PEF/1PEF ratio, *η*, both for the mutants (red solid line) and the reference (black dashed line).

Because the mutation rate in the 1^st^ round was expectedly low, approximately 1 mutation per mutant, there were only a few mutants that significantly deviated from the parent EGFP, with the majority of the red dots in Fig. 2a lining up close to *η*  =  1. The corresponding histogram plot in Fig. 2b and Supplementary Fig. 2 shows that in about 99% of cases *η*  <  1.3, i.e. the mutants were virtually indistinguishable from the parent EGFP. However, among the remaining population there were about 100 colonies (out of the ~7,500 colonies in the 1^st^ library) that showed a potentially enhanced 2PEF/1PEF ratio, and thus lied above the *η*  =  1.3 line (solid black line in Fig. 2a). 59 of these colonies were picked and subjected to further error prone PCR and gene shuffling that created the 2^nd^ generation mutagenized library. Figure 2c shows that in the 2^nd^ library more of the red dots are shifted above the dashed diagonal line indicating that the 2^nd^ generation of mutants has a much larger population of colonies with useful mutations. The corresponding histogram (Fig. 2d and Supplementary Fig. 2) shows that ~42% of the colonies have *η*  >  1.3.

The increase of *η* may follow either from an increase of the two-photon cross section at λ_2PA_ or from a decrease of the one-photon cross section at λ_1PA_ (or both). The fact that most of the 2^nd^ round mutants in Fig. 2c show a substantial increase of *η* points in the direction of a shifting or changing 1PA spectrum, which is accompanying the change of σ_*2PA*_. It is also interesting to note that on the scatter graph the mutants appear to congregate in distinct groups that have similar *η*. To verify this observation, we performed DNA sequencing of ~10 colonies picked from each of the ten groups (total of 96 colonies) and confirmed that each group indeed corresponds to a particular mutation.

Among the total 12 unique mutants found in the 2^nd^ round, one labeled 2.18.01 (indicated by an arrow in Fig. 2c) featured the chromophore structure altering mutation T65S, and was thus excluded from further rounds of mutagenesis. The other 11 mutants preserved the EGFP chromophore structure, and were used as the DNA template for the 3^rd^ and final round of error prone PCR and gene shuffling. The histogram plot in Fig. 2f and Supplementary Fig. 2 shows that about 93% of the mutagenized colonies had *η* > 1.3 in this final round. From those, 50 of the most promising colonies were picked and 11 new unique mutants were identified. The mutation type correlated well with the value of the screening parameter *η*.

Table 1 presents the peak 1PA and 2PA wavelengths and the peak 2PA cross sections of 23 unique mutants identified from the three evolution rounds. It is known that depending on the environment, GFP and related chromophores are observed in different ionization states^2^,^10^. EGFP under physiological conditions is found predominantly in the anionic form with the 1PA peak at 488 nm, and only a small fraction resides in the neutral form with the 1PA peak at 395 nm. Many of the mutants showed enhanced 1PA at shorter wavelengths, which is most likely a result of our choice of λ_2PA_ and λ_1PA_ preferring the selection of the neutral form over the ionic form. Based on this, we classified the mutants into three categories: (1) analog of the anionic form of EGFP; (2) analog of the neutral form of EGFP and (3) variants that exhibit traits of both the anionic and neutral forms of EGFP. Representative mutant 2PA and 1PA spectra from each of the three categories along with the non-mutagenized EGFP are shown in Fig. 3 (see Supplementary Information and methods for measurement details). The 1PA shape and the peak extinction coefficient of mutant 3.18 (Fig. 3a) closely resemble those of EGFP (Fig. 3d), however the 2PA peak appears to be increased by about 50% to σ_*2PA*_ = 61 GM. The other 14 mutants from the same category showed similar features, with peak 2PA values in the range of σ_*2PA*_ = 40–60 GM. In mutant 2.18.01 (Fig. 3b) the 1PA and 2PA peaks are almost entirely switched to shorter wavelengths. The 2PA spectrum closely resembles that of mAmetrine, which has a peak 2PA cross section of 56 GM at 809 nm^11^. The DNA sequence reveals the mutation T65S which is a characteristic of FPs dominated by the neutral form^10^,^25^. The spectra of mutant 3.06 (Fig. 3c) appear to be a superposition of the neutral and anionic form spectra, where the absolute cross sections of each of the forms remains hard to determine because of the unknown relative concentrations. Nevertheless, we may proceed with the comparison between different mutants if we introduce the effective 2PA cross section, σ_2PA,eff._, and the effective 1PA extinction coefficient, ε_eff._, defined respectively as:


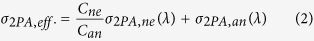



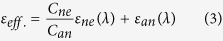


where *C*_*ne*_, and *C*_*an*_ are the relative concentration of the neutral and anionic forms, σ_*2PA,ne*_ and σ_*2PA,an*_ are the absolute two-photon cross sections of the two forms, and ε_*ne*_ and ε_*an*_ are the respective one-photon molar extinction coefficients. From our spectroscopic data we can calculate the effective cross sections if we assume that the one-photon extinction coefficient of the anionic form is equivalent to EGFP, ε_*an*_ = 55,000 M^−1^ cm^−1^ (see Methods and Supplementary Fig. 3), and that, at 950 nm the σ_*2PA,ne*_ is virtually zero, leaving only the anionic contribution to the two-photon cross section.

The mutation T203I is associated with intra-chromophore charge transfer due to breaking of a hydrogen bond with the β barrel^6^,^10^,^25^ and is likely responsible for the increase of the neutral form in all but one of the mutants from the 3^rd^ category (3.06, 3.12, 3.15, 3.43, 3.04, 2.59.01, and 2.59.08). The V163A mutation, previously reported to accelerate the protein folding^25^, is present in 17 mutants including 2.59.38, where the V163A mutation led to a 30% increase in the peak σ_*2PA*_ and a 20% increase in the relative brightness of the anionic form of the chromophore.

Further comparative inspection of the σ_2PA_ spectra in Fig. 3 reveals that in the S_0_ → S_1_ transition region 3.18 exhibits a distinct vibronic structure where the 0–1 peak prevails over the 0–0 component. This behavior has been previously observed in several FPs and chromophores and is most likely related to the Herzberg-Teller type coupling between the vibrational motion and the permanent dipole moment change (Δμ) in the S_0_ → S_1_ electronic transition of the chromophore^26^. If the anionic chromophore possesses an active bond-length-alternating vibrational coordinate^27^, then the mutations leading to 3.18 could be linked to an increased mixing between the resonance forms^1^,^26^. These same mutations could also alter the local electric field inside the protein^11^, which in turn may cause stronger vibronic coupling and therefore increase the peak 2PA value of the 0–1 transition. This tentative explanation is further supported by the observation that most mutants in the same category as mutant 3.18 had similar 2PA values for the purely electronic 0–0 transition, 30 ± 5 GM, while the value of the vibronic 0–1 transition varied from 40 GM to 61 GM (see Supplementary Fig. 3). Table 1 lists the Δμ values in the S_0_ → S_1_ transition determined from the σ_2PA_ of the 0–0 component^28^,^29^ (see ref. 25 for calculation details).

In summary, we have developed a new high through-put *in situ* multi-photon excited fluorescence screening procedure that facilitates directed evolution of genetically encoded multi-photon probes. We put this procedure to the test by evolving new EGFP variants (V163A, Q184R and N121S, V163A) with 50% enhanced 2PA cross section and roughly 50% enhanced 2PEF brightness at a near-IR excitation wavelength. Our method can be used to optimize the two-photon and higher-order multi-photon properties of many different FP types, especially if combined with an appropriately tunable wavelength excitation source. Developing a broad color palette of efficient multi-photon probes may become instrumental for imaging of the brain and other complex tissues that comprise many different cell types^3^,^9^,^15^,^17^,^30^,^31^,^32^. By tuning both the λ_2PA_ and λ_1PA_ to the 0–0 component of the S_0_ → S_1_ transition of the chromophore, we can potentially maximize the voltage sensitivity of FPs by increasing the change of the permanent eclectic dipole moment, |Δμ|. Already at the current level of accuracy, this procedure enables a rather detailed quantitative comparison of mutant FPs not only from different Petri plates, but also across different libraries measured under different conditions. We expect that future experiments will facilitate the establishment of practical structure-property relationships between different mutation types and their corresponding multi-photon properties.

## Methods

Additional methods are provided in the Supplementary Information.

### Measurement of the 2PEF and 1PEF signals

**Figure 1:**
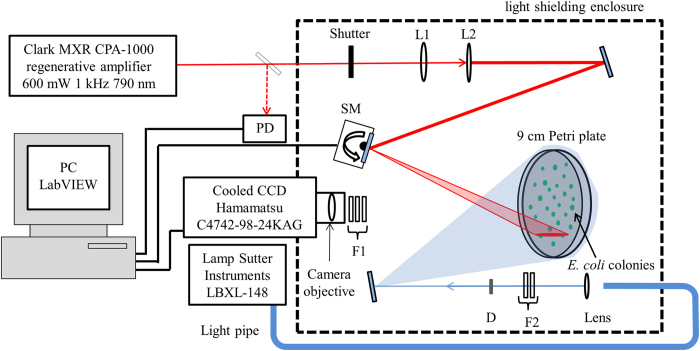
Schematic of the 2PEF and 1PEF imaging setup. L1, cylindrical lens; L2, spherical lens; PD, photo diode; SM, scanning mirror; F1, fluorescence detection filters; F2, one-photon excitation wavelength selection filters; D, diffuser.

For verification that the 2PEF image represents the true 2PEF of the sample the exponent of the measured fluorescent signal’s power dependence was measured and found to be 2.0 ± 0.01^33^. The Hamamatsu C474-98-24KAG cooled CCD camera and Petri plate were enclosed in a semi-light-tight box and the room lights were shut off to reduce background light. A stack of color filters with a center wavelength of 535 nm and 40 nm band pass were used in front of the CCD camera (F1 in Fig. 1) to select for the desired fluorescence emission wavelength and block any scattered excitation light. A combination of a band pass filter with a center wavelength of 450 nm and a band pass of 50 nm and a neutral density filter (F2 in Fig. 1) were used in front of the lamp source to select for the one-photon excitation wavelength and to adjust the excitation photon flux. A glass diffuser plate (D in Fig. 1) was used in front of the lamp source to provide spatially uniform intensity on the sample. A PC with Windows XP was used to control the camera, monitor the laser power, and control the Zaber T-OMG motorized x-y-axis optical mount through a custom program written in LabVIEW. In order to quantitatively compare the fluorescence images from different Petri plates in a library the illumination intensity each Petri plate receives should be relatively similar. To accomplishing this goal the laser and lamp were allowed sufficient time to reach an equilibrium temperature before each measurement was made. Before each library was scanned, the laser system was allowed to warm up for at least one hour. The camera was also given some time to reach its equilibrium CCD temperature of –30 °C. While the laser was warming up, the first Petri plate was inserted into a custom built mounting plate that holds the Petri plate vertically with the *E. coli* colonies facing the camera. The LabVIEW program was initialized, and the Sutter Instruments LBXL-148 lamp source was turned on and allowed to warm up. While the lamp was reaching its optimal operating temperature, the first set of images was taken. The sample was illuminated by white light to capture a scattered light image showing the position of all of the bacterial colonies on the Petri plate including non-fluorescent colonies. Once the lamp source was at its optimal operating temperature, four images of the 1PEF were captured, averaged together in LabVIEW, and saved. For all images captured, both 1PEF and 2PEF, an 8 second exposure time was used. To measure the dark background, the lamp was shut off and four images with no illumination (both the lamp and laser were blocked) were averaged together and saved. These images were used to subtract any background signal from all of the fluorescence images. Finally, the shutter blocking the laser was opened and the 2PEF was collected. This was done by scanning the laser illumination across the Petri plate vertically (y axis in Supplementary Fig. 4b) once per image and stepping the horizontal (x axis in Supplementary Fig. 4b) position of the laser stripe after each image. In our measurement of mutagenized EGFP libraries a total of 60 horizontal steps and two laser passes (two images) per step were taken, resulting in 112 total images being used to calculate the final 2PEF image for each Petri plate. Measuring the 2PEF of one plate took approximately 20 minutes. Averaging of the images was done in LabVIEW such that the final output was a single image of the 2PEF. During the collection of the 2PEF image, the laser power as a function of position of the vertical illumination stripe on the Petri plate was recorded, using a Molectron P4-35 power detector, and saved. These steps were repeated for each Petri plate in the library being scanned. Collected data were imported into a MATLAB program that calculates the 2PEF and 1PEF signals of each colony and evaluates the 2PEF versus 1PEF ratio. Neither the camera nor the samples were disturbed while the 2PEF and 1PEF images were captured which ensured both final images overlapped exactly with each other facilitating quantitative comparison of the 1PEF and 2PEF fluorescence data. The estimated spatial resolution of the imaging setup is 7 lines per mm.

### Measuring the two-photon absorption cross section

The 2PA spectra were measured using the fluorescence excitation method described in detail in^34^. The 2PA spectra of all of the mutants selected from the three mutagenized libraries were measured with respect to the reference standard fluorescein in pH11 buffer solution. The quadratic power dependence of the 2PEF signal was checked at either 770 nm and 930 nm or 750 nm and 950 nm. The exponent of the power dependence measurement was 2.0 ± 0.05. The one-photon absorption (1PA) spectra were measured using either a Perkin Elmer Lambda 950 or a Perkin Elmer Lambda 900 spectrometer. The quantum yields were measured by comparing the ratio of the fluorescence and optical density of the samples to that of fluorescein. The fluorescence spectra were measured using a Perkin Elmer LS 50 B spectrofluorometer. The one-photon extinction coefficients were measured for a select number of mutants using the alkaline denaturation method^35^. Extinction coefficients of the anionic form of the chromophore for all mutants measured were found to be 55 ± 6 × 10^3^ M^−1^ cm^−1^. A value of 55 × 10^3^ M^−1^ cm^−1^ for the extinction coefficient of the anionic form of the chromophore was used for all calculations of the two-photon cross section.

## Additional Information

**How to cite this article**: Stoltzfus, C. R. *et al.* Two-photon directed evolution of green fluorescent proteins. *Sci. Rep.* **5**, 11968; doi: 10.1038/srep11968 (2015).

## Supplementary Material

Supplementary Information

## Figures and Tables

**Figure 2:**
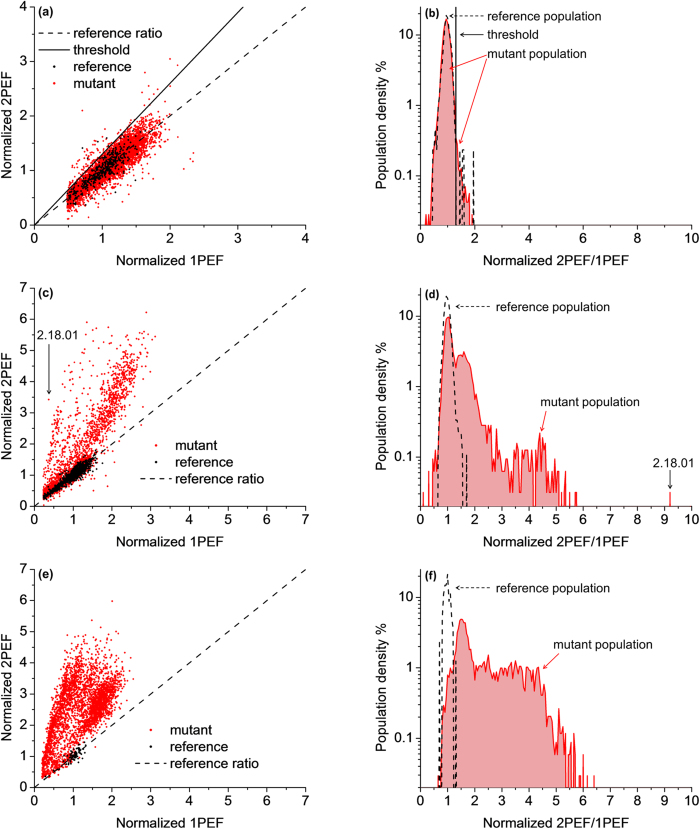
Fluorescence data of randomly mutagenized EGFP. Left panel: Normalized 2PEF signal plotted versus normalized 1PEF signal of the mutagenized (red symbols) and non-mutagenized (black symbols) colonies. Each point represents a single colony. The black dashed line corresponds to *η*  = 1, the average slope of non-mutated EGFP colonies. Right panel: Histogram representation of the data shown in the left panel in terms of percentage of the colonies (vertical axis) with a particular ratio value *η* (horizontal axis). (**a, b**), The 1^st^ generation library. The black solid line corresponds to *η*  = 1.3. Mutants that appear above this line were used as the DNA template for the second library. (**c, d**), The 2^nd^ generation library. Black arrows highlight the normalized integrated fluorescence and the normalized ratio of the colony expressing mutant 2.18.01. (**e, f**), The 3^rd^ generation mutagenized library. The 1^st^ 2^nd^ and 3^rd^ libraries contained 7,536, 3,192, and 3,423 colonies respectively. Colonies that could not be reliably identified, e.g. due to low fluorescence signal, spatial overlap between neighboring colonies, or close proximity to the Petri plate’s outer rim, were eliminated from consideration (see Methods and Supplementary Information).

**Figure 3:**
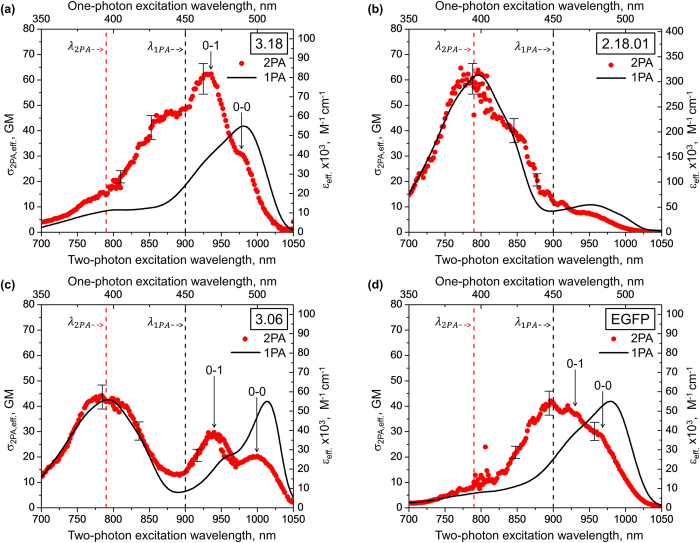
Absorption spectra of selected mutants of EGFP. Two-photon absorption cross section (red symbols) with 10% error (black error bars) and one-photon extinction (black solid lines) of the selected representative mutants and EGFP. Vertical dashed red and black lines represent λ_2PA_ and λ_1PA_, respectively. The vertical arrows indicate the peak wavelengths of the vibronic components. The 2PA spectra of all 23 mutants are presented in Supplementary Fig. 3. The numbers in the upper right hand corner of each plot designate different mutants.

**Table 1:** Photophysical properties and DNA analysis data of the 23 selected mutants of EGFP.

Group	FP	Mutations	Anionic form σ_2PA,an_ GM (λ_max_ nm)	Neutral form 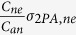 GM (λ_max_ nm)	Anionic form 1PA λ_max_ nm	Anionic form Relative Brightness	Δµ(S_0_ → S_1_) D
**1**	**EGFP**		**42 (896)**	**11 (806)**	**488**	**1.0**	**3.5**
**1**	**3.18**	**V163A, Q184R**	**61 (936)**	**18 (806)**	**491**	**1.4**	**2.5**
**3**	**2.59.17**	**N121S, V163A**	**61 (910)**	**33 (788)**	**488**	**1.5**	**3.1**
**1**	**3.01**	**S72G**	**60 (932)**	**31 (806)**	**495**	**1.2**	**3.5**
**1**	**3.26**	**D117G, V163A**	**60 (928)**	**17 (798)**	**491**	**1.4**	**2.3**
**1**	**3.21**	**V68M, V163A, extra A.A. C-term**	**57 (932)**	**33 (798)**	**492**	**1.3**	**2.3**
**1**	**3.02**	**S72G, Q184R**	**56 (934)**	**27 (806)**	**495**	**1.1**	**3.4**
**1**	**2.59.14**	**V68M**	**54 (912)**	**31 (788)**	**490**	**1.3**	**3.0**
**1**	**2.59.38**	**V163A**	**54 (902)**	**22 (812)**	**491**	**1.2**	**3.1**
**1**	**2.18.13**	**D117G, V163A, S202N, V219I**	**53 (904)**	**28 (826)**	**492**	**1.2**	**3.0**
**1**	**3.30**	**S72G, D117G, V163A**	**52 (934)**	**26 (800)**	**498**	**1.0**	**2.5**
**1**	**2.18.08**	**V68M, V163A**	**51 (898)**	**31 (808)**	**492**	**1.2**	**2.9**
**1**	**2.18.15**	**V163A, S202N**	**46 (912)**	**17 (788)**	**491**	**1.0**	**2.7**
**1**	**2.59.12**	**V68M, N105S, V163A**	**44 (910)**	**31 (788)**	**492**	**0.90**	**2.7**
**1**	**2.18.12**	**E6G, S72G, V163A**	**42 (906)**	**32 (808)**	**498**	**0.80**	**2.9**
**1**	**2.18.19**	**S72G, V163A**	**40 (900)**	**27 (792)**	**498**	**0.76**	**2.9**
**3**	**3.06**	**D117G, V163A, T203I**	**33 (946)**	**47 (784)**	**507**	**0.88**	**3.4**
**3**	**3.12**	**Q80R, D117G, V163A, T203I**	**35 (942)**	**46 (806)**	**507**	**0.80**	**3.3**
**3**	**3.15**	**S72G, T203I**	**35 (940)**	**32 (782)**	**506**	**0.68**	**2.7**
**3**	**3.43**	**E6G, S72G, V163A, T203I**	**35 (920)**	**25 (798)**	**507**	**0.79**	
**3**	**3.04**	**V68M, V163A, T203I**	**27 (946)**	**59 (784)**	**507**	**0.68**	**2.8**
**3**	**2.59.01**	**V163A, T203I**	**23 (952)**	**48 (790)**	**508**	**0.51**	**2.6**
**3**	**2.59.08**	**T203I**	**19 (948)**	**48 (790)**	**507**	**0.44**	**2.4**
**2**	**2.18.01**	**T65S, S202N**	**8 (940)**	**61 (772)**	**476**	**0.39**	

The first column on the left shows the correspondence to one of the 3 groups. In the second and third column is the name of the mutant and identity of the mutations from the DNA analysis. In the fourth and fifth columns are the peak 2PA cross sections of the anionic form and the effective peak 2PA cross section of the neutral form along with the corresponding wavelengths (in parentheses). In the sixth column is the peak 1PA wavelength of the anionic form. The last two columns on the right show the brightness of the peak 2PEF, defined as σ_2PA,an*_ ϕ_*rel*_ (see Supplementary Information), of the anionic form of the chromophore relative to EGFP and the permanent electric dipole moment change in the S_0_ → S_1_ transition.

## References

[b1] Bell A. F., He X., Wachter R. M. & Tonge P. J. Probing the ground state structure of the green fluorescent protein chromophore using Raman spectroscopy. Biochemistry 39, 4423–4431 (2000). 10757992 10.1021/bi992675o

[b2] Chattoraj M., King B. A., Bublitz G. U. & Boxer S. G. Ultra-fast excited state dynamics in green fluorescent protein: multiple states and proton transfer. Proc Natl Acad Sci USA 93, 8362–8367 (1996). 8710876 10.1073/pnas.93.16.8362 PMC38676

[b3] Chudakov D. M., Matz M. V., Lukyanov S. & Lukyanov K. A. Fluorescent proteins and their applications in imaging living cells and tissues. Physiol Rev 90, 1103–1163 (2010). 20664080 10.1152/physrev.00038.2009

[b4] Hanson G. T. *et al.* Green fluorescent protein variants as ratiometric dual emission pH sensors. 1. Structural characterization and preliminary application. Biochemistry 41, 15477–15488 (2002). 12501176 10.1021/bi026609p

[b5] Heim R., Cubitt A. B. & Tsien R. Y. Improved green fluorescence. Nature 373, 663–664 (1995). 7854443 10.1038/373663b0

[b6] Heim R., Prasher D. C. & Tsien R. Y. Wavelength mutations and posttranslational autoxidation of green fluorescent protein. Proc Natl Acad Sci USA 91, 12501–12504 (1994). 7809066 10.1073/pnas.91.26.12501 PMC45466

[b7] Patterson G. H., Knobel S. M., Sharif W. D., Kain S. R. & Piston D. W. Use of the green fluorescent protein and its mutants in quantitative fluorescence microscopy. Biophys J 73, 2782–2790 (1997). 9370472 10.1016/S0006-3495(97)78307-3 PMC1181180

[b8] Peter M. *et al.* Multiphoton-FLIM quantification of the EGFP-mRFP1 FRET pair for localization of membrane receptor-kinase interactions. Biophys J 88, 1224–1237 (2005). 15531633 10.1529/biophysj.104.050153 PMC1305125

[b9] Svoboda K. & Yasuda R. Principles of two-photon excitation microscopy and its applications to neuroscience. Neuron 50, 823–839 (2006). 16772166 10.1016/j.neuron.2006.05.019

[b10] Tsien R. Y. The green fluorescent protein. Annu Rev Biochem 67, 509–544 (1998). 9759496 10.1146/annurev.biochem.67.1.509

[b11] Drobizhev M., Makarov N. S., Tillo S. E., Hughes T. E. & Rebane A. Two-photon absorption properties of fluorescent proteins. Nat Methods 8, 393–399 (2011). 21527931 10.1038/nmeth.1596 PMC4772972

[b12] Rajul K. & Jain R. R. Local complexity of amino acid interactions in a protein core. Proc Natl Acad Sci USA 101, 111–116 (2004). 14684834 10.1073/pnas.2534352100 PMC314147

[b13] Ai H., Baird M. A., Shen Y., Davidson M. W. & Campbell R. E. Engineering and characterizing monomeric fluorescent proteins for live-cell imaging applications. Nat Protoc 9, 910–928 (2014). 24651502 10.1038/nprot.2014.054

[b14] Akerboom J. *et al.* Optimization of a GCaMP calcium indicator for neural activity imaging. Journal of Neuroscience 32, 13819–13840 (2012). 23035093 10.1523/JNEUROSCI.2601-12.2012 PMC3482105

[b15] Broussard G. J., Liang R. & Tian L. Monitoring activity in neural circuits with genetically encoded indicators. Front. Mol. Neurosci. 7, 1–17 (2014). 25538558 10.3389/fnmol.2014.00097 PMC4256991

[b16] Davidson M. W. & Campbell R. E. Engineered fluorescent proteins: innovations and applications. Nat Methods 6, 713–717 (2009). 19953681 10.1038/nmeth1009-713

[b17] Bovetti S., Moretti C. & Fellin T. Mapping brain circuit function *in vivo* using two-photon fluorescence microscopy. Microsc Res Tech 77, 492–501 (2014). 24504776 10.1002/jemt.22342

[b18] Hoover E. E. & Squier J. A. Advances in multiphoton microscopy technology. Nat Photonics 7, 93–101 (2013). 24307915 10.1038/nphoton.2012.361 PMC3846297

[b19] Liang R., Broussard G. J. & Tian L. Imaging chemical neurotransmission with genetically encoded fluorescent sensors. ACS Chem. Neurosci. 6, 84–93 (2015). 25565280 10.1021/cn500280k

[b20] Marblestone A. H. *et al.* Physical principles for scalable neural recording. Front Comput Neurosci 7, 1–34 (2013). 24187539 10.3389/fncom.2013.00137 PMC3807567

[b21] Carriles R. *et al.* Invited review article: Imaging techniques for harmonic and multiphoton absorption fluorescence microscopy. J. A. Rev. Sci. Instrum. 80, 81101 (2009). 10.1063/1.3184828 PMC2736611 19725639

[b22] Denk W., Strickler J. H., Webb W. W. Two-photon laser scanning fluorescence microscopy. Science 248, 73–76 (1990). 2321027 10.1126/science.2321027

[b23] Diaspro A., Chirico G. & Collini M. Two-photon fluorescence excitation and related techniques in biological microscopy. Q. Rev. Biophys. 38, 97–166 (2005). 16478566 10.1017/S0033583505004129

[b24] Elowitz M. B., Levine A. J., Siggia E. D. & Swain P. S. Stochastic gene expression in a single cell. Science 297, 1183–1186 (2002). 12183631 10.1126/science.1070919

[b25] Cubitt A. B., Woollenweber L. A. & Heim R. Understanding structure-function relationships in the Aequorea victoria green fluorescent protein. Methods Cell Biol 58, 19–30 (1999). 9891372 10.1016/s0091-679x(08)61946-9

[b26] Drobizhev M., Makarov N., Tillo S., Hughes T. & Rebane A. Describing two-photon absorptivity of fluorescent proteins with a new vibronic coupling mechanism. J Phys Chem B 116, 1736–1744 (2012). 22224830 10.1021/jp211020k PMC3280616

[b27] Meyers F., Marder S., Pierce B. & Bredas J. Electric field modulated nonlinear optical properties of donor-acceptor polyenes: sum-over-states investigation of the relationship between molecular polarizabilities (alpha ,beta , and gamma) and bond length alternation. J. Am. Chem. Soc. 116, 10703–10714 (1994).

[b28] Drobizhev M., Tillo S., Makarov N., Hughes T. & Rebane A. Color hues in red fluorescent proteins are due to internal quadratic stark effect. J. Phys. Chem. B 113, 12860–12864 (2009). 19775174 10.1021/jp907085p PMC2893592

[b29] Rebane A. *et al.* Quantitative Prediction of Two-Photon Absorption Cross Section Based on Linear Spectroscopic Properties†. J Phys Chem C 112, 7997–8004 (2008).

[b30] Akemann W., Mutoh H., Perron A., Rossier J. & Knöpfel T. Imaging brain electric signals with genetically targeted voltage-sensitive fluorescent proteins. Nat Methods 7, 643–649 (2010). 20622860 10.1038/nmeth.1479

[b31] Akerboom J. *et al.* Genetically encoded calcium indicators for multi-color neural activity imaging and combination with optogenetics. Front Mol Neurosci 6, 1–29 (2013). 10.3389/fnmol.2013.00002 PMC3586699 23459413

[b32] Tillo S. E., Hughes T. E., Makarov N. S., Rebane A. & Drobizhev M. A new approach to dual-color two-photon microscopy with fluorescent proteins. BMC Biotechnol 10, 6 (2010). 20122267 10.1186/1472-6750-10-6 PMC2831818

[b33] Stoltzfus C. *et al.* A multidimensional screening method for the selection of two-photon enhanced fluorescent proteins. Proc SPIE 8956, 895611–895611 (2014).

[b34] Makarov N. S., Drobizhev M. & Rebane A. Two-photon absorption standards in the 550-1600 nm excitation wavelength range. Opt Express 16, 4029–4047 (2008). 18542501 10.1364/oe.16.004029

[b35] Ward W. W. Biochemical and physical properties of green fluorescent protein. Green fluorescent protein: properties, applications and protocols. 2nd ed. Hoboken, USA: Wiley and Sons 39–65 (2005). 16335709

