# Supplementary material for: Two-photon directed evolution of green fluorescent proteins
Source: Sci Rep. 2015 Jul 6;5:11968. doi: 10.1038/srep11968 (PMC4491718; doi:10.1038/srep11968)
Supplement: Supplementary Information [file srep11968-s1.pdf]

# Two-photon Directed evolution of green fluorescent proteins

Caleb R. Stoltzfus[a], Lauren M. Barnett[b], Mikhail Drobizhev[a], Geoffrey Wicks[a], Alexander Mikhaylov[a], Thomas E. Hughes[b], Aleksander Rebane[a,c,*]

[a]Physics Department, Montana State University, Bozeman MT. 59717; [b]Cell Biology Neuroscience Department, Montana State University, Bozeman MT. 59717, [c]National Institute of Chemical Physics and Biophysics, Tallinn, Estonia, 12618,

*Corresponding author

## Supplementary Information

**Analysis of the 2PEF and 1PEF images.**

Once the data were collected for each library, the digital images of the 1PEF, 2PEF, and background were imported into a custom program written in MATLAB. The program first subtracts the background image for each Petri plate from the corresponding fluorescence images to account for scattered ambient light and noise in the camera. The 1PEF image is corrected for variations in the distribution of the lamp source illumination intensity and the 2PEF image is corrected for changes in laser power throughout the scan. The lamp source illumination variation was measured by recording the average 1PEF from a piece of uniformly fluorescent paper placed in a Petri plate and held in the Petri plate holder in the imaging setup. The paper should be placed on top of a piece of cardboard to assure that it is at the same height in the Petri plate as the *E. coli* colonies in a typical library. This average 1PEF as a function of position in a Petri plate was normalized to its peak value and any pixels outside the Petri plate in the resulting averaged 1PEF image were set to 1 such that nothing was divided by 0. The 1PEF images of the Petri plates containing the mutagenized FP expressing *E. coli* colonies can then be divided by this normalized image. Our correction factor ranged from 1 at the center of the 1PEF normalization image to 0.2 at the edge of the Petri plate. For the three libraries in the validation study, no corrections to the 2PEF images due to laser power variations were necessary. If the laser power does change over the course of the measurement of the 2PEF image this can be accounted for by multiplying each column of the image (if the laser stripe is scanned vertically as it was here) by a correction factor based on the normalized laser power recorded while that column of the image was being illuminated. When corrections must be done to the 2PEF image it is important to take into account the fact that the 2PEF depends on the square of the laser power. These corrections are necessary to assure accurate comparison of the signals from different *E. coli* colonies in different positions on a given Petri plate. Unfortunately, agar at the edge of most Petri plates climbs up the side of the plate resulting in odd artifacts in the edge of the fluorescence images. To deal

with this problem, any pixels within 2 mm of the edge of the Petri plate were set to zero. The total fluorescence of each colony was found by identifying the brightest pixel in the 1PEF image, recording the position of that pixel, saving the sum of the pixel values for an area around the selected pixel in both the 1PEF and 2PEF images, and then setting that area to zero in the original image such that the next brightest pixel, representing the next brightest colony's fluorescence signal, could be found. The size of the area that is summed depends on how large the colonies were allowed to grow and the camera setup used. Typically this is set once for each library to be about the size of the largest colony such that no fluorescence is missed. For the colonies we used this was typically an 11 pixel by 11 pixel area. This process was repeated until all of the fluorescing colonies were identified and their corresponding 1PEF and 2PEF signals recorded. The total 1PEF and 2PEF signals were all normalized relative to the corresponding average 1PEF or 2PEF fluorescent signal of the colonies from the reference scan.

**Demonstration of the variable absolute brightness of FP expressing *E. coli* colonies.**

Supplementary Fig. 1 shows the 2PEF (a) and 1PEF (b) images of a sample containing two kinds of *E. coli* colonies growing in particular patterns. The first type expressed the mTFP1 protein ("NIH" letter pattern in the center of the plate) and the second expressed the mWasabi protein (colonies distributed randomly across the plate). The individual colonies appear as bright spots on dark background. mTFP1 and mWasabi are known to have notably different 2PA properties at the excitation wavelength 790 nm: the 2PA cross section of mTFP1 is, $\sigma_{2PA}$(790 nm) = 10 GM, while that of mWasabi is, $\sigma_{2PA}$(790 nm) = 1 GM. Due to this difference, the mTFP1 colonies appear, in the 2PEF image (in Supplementary Fig. 1a) on average as brighter spots than the corresponding mWasabi colonies. At the same time, the 1PA cross section and the 1PEF brightness of the two types of FPs is comparable. Accordingly, in Supplementary Fig. 1b both types show similar fluorescence signals.

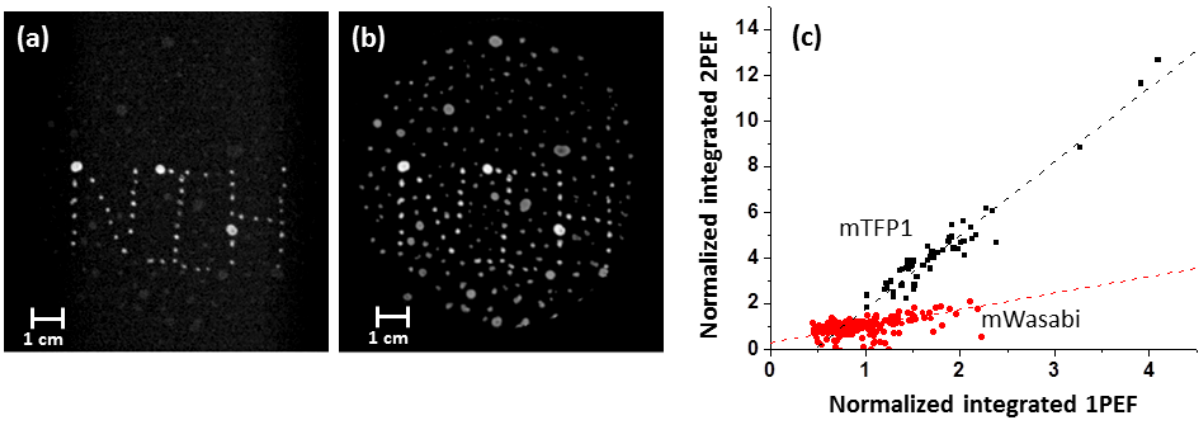


**Supplementary Figure 1| Fluorescence images of a Petri plate with intermixed *E. coli* colonies.** Colonies are expressing mTFP1 ("NIH" pattern) and mWasabi (randomly distributed); (a) 2PEF image; (b) 1PEF image. (c) Normalized integrated 2PEF signal (vertical axis) from each of the mTFP1 (black symbols) and mWasabi (red symbols) colonies plotted versus the corresponding normalized integrated 1PEF signal (horizontal axis). Each point on the graph represents an individual colony of *E. coli* bacteria expressing either one or the other FP type. Dashed lines are the linear fits to the corresponding data points.

A careful inspection of Supplementary Fig. 1a and 1b reveals a fairly large variation of the colony size as well as 2PEF brightness, some of the colonies emitting several times more overall fluorescence than other colonies expressing the same type of FP. One can imagine that if we would need to differentiate between thousands of unknown mutants, where the mutations can further affect these factors, these variations would make any quantitative comparison and selection of the best two-photon mutants highly problematic. Fortunately, the ratio between the 2PEF and 1PEF signals from the same type of FP is more or less constant and appears to be unaffected by these factors. Supplementary Fig. 1c shows the correlation plot between the 2PEF signal (vertical axis) and 1PEF signal (horizontal axis) from each individual colony from Supplementary Fig. 1a and 1b. Every point on the plot represents the normalized fluorescence intensity integrated over the whole area of the corresponding colony. The data shows that nearly all colonies fall into two distinct groups according to their type and that each group approaches a distinct linear fit line. The slope of this line can serve as a practical quantitative measure of the 2PEF efficiency that is both independent of most growth-related factors, as well as reproducible from one plate to another.

**Preparation of non-mutated mixed FP plates pictured in Supplementary Fig. 1.**

For the purpose of demonstrating the differences between the 1PEF and 2PEF of different known FPs, we prepared the plates shown in Supplementary Fig. 1. The encoding regions of mWasabi and TFP1.0 were first cloned into the same plasmid used for constitutive bacterial expression in our EGFP mutant screening (pCP, from Nathan Shaner) using the same ligation-independent cloning protocol used for EGFP. *E. coli* expressing mWasabi and TFP1.0 were then manually spotted on the black LB agar plates using a thin wire into a pattern constituting either the background or the “NIH” pattern, respectively. The plates used were the same 9cm Petri plates containing black LB Agar with Ampicillin as used for the mutant EGFP libraries. This method of manually spotting the *E. coli* colonies on the plate places more than one bacterium at each position leading to greater variation in final colony size after incubation than does plating *E. coli* using glass beads, as we did with our mutant libraries. The spotted *E. coli* plates were allowed to incubate overnight at 37ºC and then scanned the next day using the same procedures used for scanning the mutated libraries.

Once the 2PEF and 1PEF images were captured and averaged in LabVIEW they were imported into a custom MATLAB program. This program first cropped the 1PEF and 2PEF images to include only the Petri plate. The images were then converted to matrices of pixel values. The 1PEF and 2PEF matrices were normalized to their maximum values. A threshold was used on both matrices such that small background pixels were set to a value of 1. This ensured that nothing was divided by 0. The 1PEF threshold was set to 0.17 and the 2PEF threshold was set to 0.09. The 2PEF matrix was divided by the 1PEF matrix. A threshold was imposed on the final matrix of ratios of $0.2<\text{matrix}<1$. All pixel values outside this range were set to 0. The 1PEF and 2PEF background pixels that were initially set to 1 in the threshold were set to 0. Finally the matrices were converted back to images and saved.

**Random mutagenesis of EGFP.**

All error-prone PCR was performed using Taq DNA Polymerase (New England BioLabs) in the presence of 0.04µM $MnCl_2$. All primers used to amplify EGFP and the mutant variants were complementary to the translation initiation and the 3' end of the EGFP sequence and included 15 base pair joints of sequence complementary to the cloning plasmid on the 5' and 3' ends for ligation-independent cloning (In-Fusion® HD, Clontech). The gene shuffling used in the PCR reactions of the second and third round of mutagenesis consisted of an initial series of brief elongation steps (6 rounds total) of increasing time (from 10sec to 60sec) before the start of 30 rounds of traditional PCR. All DNA sequencing was performed by GenScript (http://www.genscript.com).

For the first round of mutagenesis, the encoding region of EGFP was amplified using error-prone PCR. From the first round screening results, 100 colonies were selected above an arbitrary value of 2PEF/1PEF ratio.

For the second round of mutation, two reactions were used to determine the ideal DNA template for amplification. The template pool for the first reaction (named m2.59) included all 59 of the 100 colonies selected from the second screening of the first round library that reproducibly showed a larger 2PEF/1PEF ratio compared to the parent EGFP. The m2.59 reaction used only error-prone PCR. The template for the second, more exclusive reaction (m2.18) was the top 18 of the 59 reproducibly different 2PEF/1PEF ratio colonies from the second screening of the first round library. The m2.18 reaction used error-prone PCR and gene shuffling. Both libraries produced similar results, therefore 96 colonies with improved 2PEF/1PEF ratios were selected from both m2.59 and m2.18 libraries and sent off for DNA sequencing.

The 11 unique mutant GFP variants identified by DNA sequencing moved on as DNA template for the third and final round of error-prone PCR and gene shuffling.

**Cloning and Library preparation.**

In-Fusion cloning reactions (Clontech) were used to insert the randomly mutated FP coding region from the aforementioned PCR reactions into our cloning plasmid and then transform *E.coli* (dh5-α High Efficiency chemically competent cells, New England BioLabs). The cloning plasmid was selected for constitutive expression of his-tag proteins in bacteria (pCP, generous gift from Nathan Shaner). The transformation reactions were then diluted with SOC media and plated with glass beads on multiple (10-14) Petri plates (9cm) with black LB agar and Ampicillin. Black LB agar plates were made by adding Rublev Lamp Black pigment (a natural carbon pigment for artists, www.naturalpigments.com) to the LB agar. The Lamp Black agar plates significantly reduced laser scatter and background fluorescence from the LB agar. Plates were screened within 2 days of transforming *E. coli*. For each round, one plate of non-mutated parent EGFP was plated and screened under identical experimental conditions for reference. The parent EGFP reference corrected for day-to-day fluctuations in equilibrium laser power, changes in camera settings, and other variations in experimental conditions. By using a reference sample, the need to fully

characterize all of the detection and laser parameters of the system each day was avoided, and different libraries scanned under different conditions could be quantitatively compared.

**Selection and acquisition of mutated FPs.**

In all three rounds of EGFP mutation the following procedures were used to pick interesting mutants. Once the fluorescence signal was cataloged for each colony, the selection criterion to be used to screen the mutated FPs was defined. This was done using a custom MATLAB program where the user can select mutants by either setting a threshold (1.3 for the first library) for the ratio of the 2PEF and 1PEF signals or by manually selecting mutants (picked out of the apparent groups of mutants for the second and third libraries) from the correlation plot of the 2PEF versus 1PEF signals. As long as the Petri plates containing the library being analyzed are properly stored, selection of interesting mutants can be done at any time after the fluorescence signals have been recorded for as long as the *E. coli* are alive. Once the desired selection parameters are defined, the MATLAB program used generates an image showing which Petri plate each particular mutant fitting the selection criteria is from and where on that Petri plate that mutant is located. This image is used to pick the mutated colonies of interest from their respective Petri plates, label them, and keep them for use in later libraries and for further characterization. Colonies of interest were picked individually with a sterile loop and used to inoculate an overnight culture of LB broth with Ampicillin. These overnight cultures were then used to extract the plasmid DNA for each mutant using PureLink Quick Plasmid Miniprep Kit (Invitrogen).

**FP purification.**

*E. coli* colonies expressing fluorescent protein (in pCP, Nathan Shaner) were picked and grown at 34ºC for 48 hours in 125mL of Circlegrow (MP Biomedicals) and Ampicillin. The expressing *E. coli* culture was pelleted and excess Circlegrow broth was removed. *E. coli* pellets were lysed using BugBuster (Novagen) and Benzonase (Novagen). Cleared lysates were then purified using Protino Ni-TED 2000 packed columns (Macherey-Nagel). Purified fluorescent proteins were eluted in 1x Protino Ni-TED stock pH8 buffer solution.

**Calculation of the relative brightness.**

The relative brightness was found using the following two equations:

$$\varphi_{rel.} = \frac{OD_{sample}(480nm)}{Fl_{sample}(512nm)} * \frac{Fl_{fluorescein}(512nm)}{OD_{fluorescein}(480nm)}$$

$$Brightness = \frac{\sigma_{2PA,an,sample}\varphi_{rel.,sample}}{\sigma_{2PA,an,EGFP}\varphi_{rel.,EGFP}}$$

where $\varphi_{rel.}$ is the quantum yield of the sample relative to fluorescein, OD is the optical density of the sample measured using a Perkin Elmer Lambda 950 or a Perkin Elmer Lambda 900 spectrometer, Fl is the fluorescence of the sample measured using a

Perkin Elmer LS 50 B spectrofluorometer, and $\sigma_{2PA,an}$ is the two photon cross section of the anionic form of the sample as reported in Table 1.

**Derivation of the 2PEF and 1PEF signals.**

A diagram of the image plane of our wide field 2PEF imaging system is shown in Supplementary Fig. 4b. The y axis represents the vertical spatial direction, the x axis represents the horizontal direction, the Petri plate is represented by the gray circle, the FP expressing *E. coli* colonies are represented by the green circles, and the illuminating laser stripe is shown by the red circles. The 2PEF is integrated on the camera while scanning the laser illumination in the y direction from 0 to $d_y$. After one vertical pass of the laser stripe the illumination is stepped to the right and another image is captured while the laser is again scanned vertically. Images are captured at each horizontal position of the vertically scanning laser stripe until all of the colonies on the Petri plate have been illuminated. In the following derivation lower indices 1, and 2 indicate one-photon and two-photon excitation respectively. Assuming that the excitation rate is always much below the saturation limit and using the two-level approximation, the number of 2PEF photons detected by the camera, from a single Petri plate containing fluorescent colonies, integrated over the wavelength interval $\lambda_{min}$ –$\lambda_{max}$, defined by the bandwidth of the filters in front of the camera, is given by:

$$F_{2PEF} = N_2 \int_{\lambda_{min}}^{\lambda_{max}} C_{FL}\left(\lambda'\right)\varphi\left(\lambda',x,y\right)d\lambda'$$

Where $C_{FL}(\lambda)$ is the differential detection efficiency (including parameters such as the efficiency of collecting fluorescence, and efficiency of the CCD camera), $N_2$ is the total number of molecules excited, and $\varphi(\lambda, x, y)$ is the differential quantum efficiency of fluorescence as a function of position (x,y) on the Petri plate. The differential quantum efficiency of fluorescence is defined in terms of the standard quantum yield, Q, by the following equation.

$$\int_0^{\infty}\varphi\left(\lambda\right)d\lambda = Q$$

The standard quantum yield is equal to the ratio of the radiative decay rate to the total decay rate of the $S_1$ sate. Assuming that the scanning laser stripe is essentially stationary on the timescale of the femtosecond pulse, the number of molecules excited per laser pulse is given by:

$$N_2 = \frac{1}{2\left(h\nu_2\right)^2}\int\int_{-\infty}^{\infty}\int \sigma_{2PA}(x,y,\lambda_{2PA})c(x,y)l(x,y)\mathrm{I}_{laser}^2(x,y,t)dxdydt$$

Where $h$ is Plank's constant, $\nu_2$ is the laser frequency, $\sigma_{2PA}$ (x,y,$\lambda_{2PA}$) is the two-photon cross section as a function of position on the Petri plate being illuminated with excitation wavelength, $\lambda_{2PA}$, c(x,y) is the concentration of FP molecules present as a

function of position on the Petri plate, $l$(x,y) is the thickness of any bacterial colonies present as a function of position on the plate, and $I_{laser}$ is the intensity of the laser pulse.

With a pulse repetition rate of $g$, a vertical scan distance of $d_v$, a camera exposure time of $\tau_{2PEF}$, and treating the pulses as delta functions in time on the time scale of the vertical scan rate, $d_v/\tau_{2PEF}$, we find that the illuminating laser stripe moves $d_v/g\tau_{2PEF}$ between each pulse. The number of molecules excited in the n$^{th}$ fluorescent colony on the Petri plate, during the k$^{th}$ vertical scan of the laser is therefore given by the following summation over all pulses in that scan:

$$N_{2,n,k} = \sum_{i=0}^{g\tau_{2PEF}} \frac{\sigma_{2PA,n}\left(\lambda_{2PA}\right)c_n}{2\left(h\nu_2\right)^2} \iiint_{-\infty}^{\infty} l(x,y)\mathrm{I}_{laser}^2(x, y-\frac{d_v}{g\tau_{2PEF}}i, t)dxdydt$$

Now that we have the number of molecules excited in the n$^{th}$ colony per vertical scan of the laser we can write down the total number of molecules excited in that colony after a full scan of the Petri plate, $N_{2,n}$.

$$N_{2,n} = \sum_{k=0}^{S_h}\sum_{i=0}^{g\tau_{2PEF}} \frac{n_v\sigma_{2PA,n}\left(\lambda_{2PA}\right)c_n}{2\left(h\nu_2\right)^2} \iiint_{-\infty}^{\infty} l(x,y)\mathrm{I}_{laser}^2(x-\frac{d_h}{S_h}k, y-\frac{d_v}{g\tau_{2PEF}}i, t)dxdydt \tag{1}$$

Here $S_h$ is defined as the number of horizontal steps of the scanning laser stripe taken, $n_v$ is the number of vertical passes of the laser stripe per horizontal step, and $d_h/S_h$ is the size of the horizontal steps taken. These parameters are defined by the LabVIEW program controlling the scanning mirror mount. Assuming the temporal and spatial profiles of the laser stripe are well described by Gaussians we can write the spatial distribution of the laser intensity as:

$$\mathrm{I}_{laser} = I_{laser}^{(0)} e^{-4\ln(2)\frac{t^2}{\tau^2}} e^{-4\ln(2)\frac{y^2}{\Delta y^2}} e^{-4\ln(2)\frac{x^2}{\Delta x^2}} \tag{2}$$

Where $\mathrm{I}_{laser}^{(0)}$ is the peak laser intensity (mJ cm$^{-2}$ s$^{-1}$), $\tau$ is the temporal pulse width (FWHM), $\Delta y$ is the vertical spatial beam width (FWHM), and $\Delta x$ is the horizontal spatial beam width (FWHM). Assuming that each colony of bacteria is spherically symmetric, with a diameter of $\Delta r_n$ (FWHM), a maximum height of $l_{0,n}$, position on the Petri plate of ($x_0$, $y_0$), and can be described by Gaussian functions, we can define the thickness of the n$^{th}$ colony as:

$$l\left(x,y\right) = l_0 e^{-4\ln(2)\frac{(x-x_0)^2+(y-y_0)^2}{\Delta r^2}} \tag{3}$$

Using this definition of the thickness of the n$^{th}$ colony, we can find the volume of that colony, $V_n$, using:

$$V_n = \iint_{-\infty}^{\infty} I\left(x,y\right)dx\,dy = \iint_{-\infty}^{\infty} I_0 e^{-4\ln(2)\frac{(x-x_0)^2+(y-y_0)^2}{\Delta r^2}} dxdy = \frac{I_{0,n}\Delta r_n^2 \pi}{\ln(16)} \quad (4)$$

Using equations (2), (3), and (4) we can solve the integral in equation (1) and write the number of molecules excited as:

$$N_{2,n} = \sigma_{2PA,n}\left(\lambda_{2PA}\right)c_n \tau_{2PEF} V_n (I_{laser}^{(0)})^2 \sum_{k=0}^{S_h}\sum_{i=0}^{g\tau_{2PEF}} \frac{\tau n_v}{\tau_{2PEF}\, 2\left(h\nu_2\right)^2}\sqrt{\frac{\pi}{2\ln(16)}}\frac{e^{-8\ln(2)\left(\frac{\left(\frac{kd_h}{S_h}-x_0\right)^2}{2\Delta r_n^2+\Delta x^2}+\frac{\left(\frac{id_v}{g\tau_{2PEF}}-y_0\right)^2}{2\Delta r_n^2+\Delta y^2}\right)}}{\sqrt{(1+\frac{2\Delta r_n^2}{\Delta x^2})(1+\frac{2\Delta r_n^2}{\Delta y^2})}}$$

If we define the function $\kappa_{scan}$ ($\Delta r_n$, $\Delta x$, $\Delta y$, $S_h$, $\tau$, $g$, $\tau_{2PEF}$, $n_v$ $d_h$, $d_v$, $x_0$, $y_0$) as the summation that takes into account all of the laser parameters and the stripe scanning parameters defined by the user in the LabVIEW program controlling the scanning mirror mount, we can write the equation for the 2PEF of the n$^{th}$ colony of bacteria on the Petri plate as:

$$F_{2PEF}(n) = \sigma_{2PA,n}\left(\lambda_{2PA}\right)c_n V_n (I_{laser}^{(0)})^2 \kappa_{scan}\int_{\lambda_{min}}^{\lambda_{max}} C_{FL}(\lambda')\varphi_n\left(\lambda'\right)d\lambda'$$

For the sake of simplicity we will assume that the differential detection efficiency and differential quantum yield is approximately constant over the bandwidth of the detection wavelengths. If we define $\lambda_{cw}$ to be the center wavelength of the detection filters in front of the camera and $\Delta\lambda$ to be the bandwidth of the detection filters, we can write:

$$\int_{\lambda_{min}}^{\lambda_{max}} C_{FL}(\lambda')\varphi_n\left(\lambda'\right)d\lambda' \approx C_{FL}(\lambda_{cw})\varphi_n\left(\lambda_{cw}\right)\Delta\lambda \quad (5)$$

$$F_{2PEF}(n) = C_{FL}(\lambda_{cw})V_n c_n \sigma_{2PA,n}\left(\lambda_{2PA}\right)\varphi_n\left(\lambda_{cw}\right)\Delta\lambda\kappa_{scan}(I_{laser}^{(0)})^2$$

We note that the parameter $C_{FL}$ that takes into account all of the detection efficiency parameters and the parameter $\kappa_{scan}$ that takes into account the laser parameters, are both constant from colony to colony on a given Petri plate. We can define $\varphi_n = \varphi_n(\lambda_{cw})\Delta\lambda$ which allows us to express the 2PEF as:

$$F_{2PEF}(n) = C_{FL}V_n c_n \sigma_{2,n}\left(\lambda_{2PA}\right)\varphi_n \kappa_{scan}(I_{laser}^{(0)})^2$$

In a similar fashion we can find the 1PEF. We will start from the equation for the 1PEF of the entire Petri plate, given by:

$$F_{1PEF} = N_1 \tau_{1PEF} \int_{\lambda_{min}}^{\lambda_{max}} C_{FL}\left(\lambda'\right) \varphi\left(\lambda', x, y\right) d\lambda'$$

Where $N_1$ is the number of molecules excited per unit time and $\tau_{1PEF}$ is the exposure time of the camera. Note that $\eta(\lambda)$, and $\varphi(\lambda, x, y)$, will be the same functions as in the 2PEF case. The number of molecules excited per unit time by one-photon absorption is given by:

$$N_1 = \iint_{-\infty}^{\infty} 3.82x10^{-21} \varepsilon(x, y, \lambda_{1PA}) c(x, y) l(x, y) \frac{I_{lamp}}{h\nu_1}(x, y) dxdy$$

Where $\varepsilon(x,y,\lambda_{1PA})$ is the one-photon extinction coefficient as a function of position on the Petri plate and one-photon excitation wavelength $\lambda_{1PA}$. The one-photon extinction coefficient is related to the one-photon cross section by: $\varepsilon=3.82*10^{-21}\ \sigma_{1PA}$. c(x,y) and $l$(x,y) represent the same concentration of FPs and thickness of the colony of bacteria as before, $I_{lamp}$ represents the intensity of the lamp source, and $\nu_1$ is the lamp frequency. Under lamp illumination the illumination light is fixed with respect to the Petri plate and can be considered constant over the area of a single colony. Using the function for the thickness of the $n^{th}$ colony we defined in equation (3), we can write down the number of molecules excited per unit time, $N_1$.

$$N_1 = 3.82x10^{-21} \varepsilon_n(\lambda_{1PA}) c_n \frac{I_{lamp}}{h\nu_1} \iint_{-\infty}^{\infty} l_0 e^{-4\ln(2)\frac{(x-x_0)^2+(y-y_0)^2}{\Delta r^2}} dxdy \qquad (6)$$

By substituting equations (4) and (5) into Equation (6) we find that the 1PEF for the $n^{th}$ colony is given by:

$$F_{1PEF}(n) = 3.82x10^{-21} \tau_{1PEF} C_{FL}(\lambda_{cw}) V_n c_n \varepsilon_n(\lambda_{1PA}) \Delta\lambda \varphi_n\left(\lambda_{cw}\right) \frac{I_{lamp}}{h\nu_1}$$

We can absorb some parameters into the constant $C_{FL}$ that now takes into account all of the camera settings, multiplicative factors and detection efficiency parameters that are constant from colony to colony on a given Petri plate to write:

$$F_{1PEF}(n) = C_{FL} V_n c_n \varepsilon_n(\lambda_{1PA}) \varphi_n I_{lamp} \tau_{1PEF}$$

In our experiment we take special care that the excitation and the fluorescence detection conditions remain constant during both 1PEF and 2PEF signal collection. This allows us to make good use of a reference sample, consisting of a Petri plate

with *E. coli* colonies expressing a non-mutated FP such as EGFP. A fresh reference sample is prepared along with each new library. We normalize both the 1PEF and 2PEF signals from each mutagenized library relative to the corresponding average 1PEF and 2PEF signals obtained from the reference sample under identical conditions. Normalization of the 1PEF and 2PEF signals makes comparison of mutant FPs to the non-mutated FP being evolved much simpler. The normalized integrated 1PEF signal may be expressed as:

$$F_{1PEF}^{norm}(n) = \frac{F_{1PEF}(n)}{C_{FL}\varepsilon_{ref}(\lambda_{1PA})\varphi_{ref}\left\langle V_r c_r \right\rangle_{ref} I_{lamp}\tau_{1PEF}} = C_{1PEF}^{ref} V_n c_n \varepsilon_n(\lambda_{1PA})\varphi_n, \quad (7)$$

where <…>$_{ref}$ stands for the average over all colonies in the reference sample. The normalized integrated 2PEF signal is:

$$F_{2PEF}^{norm}(n) = \frac{F_{2PEF}(n)}{C_{FL}\sigma_{2PA}^{ref}(\lambda_{2PA})\varphi_{ref}\left\langle V_r c_r \right\rangle_{ref} \kappa_{scan} I_{laser}^{2}\tau_{2PEF}} = C_{2PEF}^{ref} V_n c_n \sigma_{2PA}(\lambda_{2PA})\varphi_n, \quad (8)$$

By taking the ratio of equations (7) and (8) we end up with equation (1) from the main text:

$$\frac{F_{2PEF}^{norm}(n)}{F_{1PEF}^{norm}(n)} = \left(\frac{C_{2PEF}^{ref}}{C_{1PEF}^{ref}}\right)\frac{\sigma_{2PA,n}(\lambda_{2PA})}{\varepsilon_n(\lambda_{1PA})},$$

**Estimation of laser parameters**

Using the scanning parameters of our imaging setup we can estimate the required laser parameters needed to image a Petri plate in a reasonable amount of time. We will assume the following criteria are needed for realistic two-photon protein evolution: 1) the fluorescence signal measured from each colony needs to be detected with a minimum signal-to-noise ratio, S/N = 10, and 2) the screening process must have a minimum throughput of at least ten Petri plate samples per day (~1 sample per hour). We use these basic metrics, together with known general properties of FPs to evaluate what type of femtosecond laser is best suited to accomplish the named tasks.

If we write out the number of detected 2PEF photons for the n$^{th}$ colony of bacteria on a Petri plate, with its explicit dependence on all of the laser, optics, and scanning parameters we find the following equation:

$$2PEF_n = \frac{\eta_c T_f \Omega \Delta\lambda \sigma_{2PA,n}\left(\lambda_{2PA}\right) V_n c_n \varphi_n n_L^{\ 2}}{2\sqrt{2}\left(\Delta x \Delta y\right)^2 \tau}\left(\frac{\ln(16)}{\pi}\right)^{5/2}\sum_{k=0}^{S_h}\sum_{i=0}^{g\tau_{2PEF}} n_v \frac{e^{-8\ln(2)\left(\frac{\left(\frac{kd_h}{S_h}-x_0\right)^2}{2\Delta r_n^2+\Delta x^2}+\frac{\left(\frac{id_v}{g\tau_{2PEF}}-y_0\right)^2}{2\Delta r_n^2+\Delta y^2}\right)}}{\sqrt{(1+\frac{2\Delta r_n^2}{\Delta x^2})(1+\frac{2\Delta r_n^2}{\Delta y^2})}}$$

This expression is in terms of the number of photon in each pulse, $n_L$. If the number of laser pulses, g$\tau_{2PEF}$, per horizontal step of the scanning laser stripe is large we can approximate the sum over the laser pulses as an integral. This approximation holds only for high repetition rate lasers.

$$2PEF_n \approx \frac{\eta_c T_f \Omega \Delta\lambda \sigma_{2PA,n}\left(\lambda_{2PA}\right) V_n c_n \varphi_n n_L{}^2}{2\sqrt{2}\left(\Delta x \Delta y\right)^2 \tau}\left(\frac{\ln(16)}{\pi}\right)^{5/2} \sum_{k=0}^{S_h} \int_0^{g\tau_{2PEF}} n_v \frac{e^{-8\ln(2)\left(\frac{\left(\frac{kd_h}{S_h}-x_0\right)^2}{2\Delta r_n^2+\Delta x^2}+\frac{\left(\frac{id_v}{g\tau_{2PEF}}-y_0\right)^2}{2\Delta r_n^2+\Delta y^2}\right)}}{\sqrt{(1+\frac{2\Delta r_n^2}{\Delta x^2})(1+\frac{2\Delta r_n^2}{\Delta y^2})}} di$$

We will use the following typical laser and scanning parameters in our analysis of our imaging technique:

- The quantum efficiency of the camera: $\eta_c$ = 0.8%
- The transmission of the collection optics (including color filters): $T_f$ = 0.8%
- The solid angle of collection (in terms of percentage of fluorescence collected):
  - $\Omega$ = 0.001% (for our wide field two-photon imaging setup)
  - $\Omega$ = 0.14% (for a two-photon microscope with an objective with a numerical aperture of 0.7)
- Bandwidth of the detection filters: $\Delta\lambda$ = 50 nm
- Two-photon excitation wavelength: $\lambda_{2PA}$ = 790 nm
- Two-photon cross section at $\lambda_{2PA}$: $\sigma_{2PA}$ = 40 GM
- Concentration of FPs: $c_n = 5x10^{15}$, (assuming 10% of the total volume is occupied by GFP molecules)
- Differential quantum yield of fluorescence: $\varphi$ = 0.1%
- Pulse duration of the laser: $\tau$ = 150 fs (We will assume this does not change with the rep rate of the laser)
- Average laser power of the 1 kHz system: P = 0.6 W (We will vary this along with the rep rate of the laser from 0.1 W to 10 W in our analysis of this imaging technique.)
- Number of illumination photons per pulse: $n_L = \frac{P}{g}\left(\frac{hc}{\lambda_{2PA}}\right)^{-1}$
- Typical bacterial colony diameter: $\Delta r$ = 1 mm
- Bacterial colony volume: $V_n = 1.1\ mm^3$
- Vertical scan distance: $d_v$ = 100 mm
- Horizontal scan distance: $d_h$ = 100 mm
- Number of vertical laser passes at each horizontal position: $n_v$ = 2
- Position of the colony (doesn't change the measured 2PEF so put it at the center of the Petri plate): $x_0 = y_0$ = 50 mm

Now that we have defined typical values for all of the constant terms we can make some assumptions about our scan parameters to lower our number of unknowns. If we assume that the illuminating laser spot is spherically symmetric we can make the following definition:

- Laser stripe width: Δx = Δy

We also know that if we turn up the intensity of the laser much more than it is in the current setup the colonies will burn. We can thus define the optimal laser beam height using the following equation:

- Laser stripe height: $\Delta y = \sqrt{\frac{n_L}{\sqrt{C_P \tau}}}$

Here $C_P$ Is a constant that is related to the maximum laser intensity desired. For our setup this value is approximately 2.4x10^40. This should be kept below the limit of cell damage. We will assume that the Rayleigh range of the focused laser cannot be less than the thickness of the colonies of bacteria, typically ~1mm, this means the lower limit on Δy is 0.016 mm. We will also assume that we want to have a minimum signal to noise ratio of 10. If the signal to noise is limited by the camera noise we can invert equation (8) which determines the vertical scan speed, as:

- Camera noise limited camera exposure time:

$$\tau_{2PEF} = \frac{14320 d_v \sqrt{2} \left(\Delta x \Delta y\right)^2 \tau \left(\ln(16)\right)^{-5/2} \sqrt{\ln(4)\left(1+\frac{2\Delta r_n^2}{\Delta x^2}\right)}}{g\eta_c T_f \Omega \Delta\lambda \sigma_{2PA,n}\left(\lambda_{2PA}\right) V_n c_n \varphi_n n_L{}^2 \Delta y \pi^5 \left( Erf\left(\frac{2y_0\sqrt{\ln(4)}}{\sqrt{\Delta y^2 + 2\Delta r_n^2}}\right) - Erf\left(\frac{2(y_0 - d_v)\sqrt{\ln(4)}}{\sqrt{\Delta y^2 + 2\Delta r_n^2}}\right)\right)}$$

If the signal to noise ratio is limited by the laser amplification noise then the camera exposure time is determined by the number of pulses that must be averaged. This was measured for our system and occurs when the size of the vertical steps is at least 1/200$^{th}$ the size of Δy. Therefore, the camera exposure time is given by:

- Laser amplification noise limited camera exposure time: $\tau_{2PEF} = 200\frac{d_v}{g\Delta y}$

We will assume that the number of horizontal steps is such that the horizontal step size is 1/10$^{th}$ the size of $\Delta x$:

- Number of horizontal steps: $S_h = \frac{10 d_h}{\Delta x}$

We will assume that for a 1 kHz repetition rate laser the signal to noise ratio is limited by the laser amplification process and is a measurable quantity that is independent of average laser power. To measure this quantity, for the laser used in our system, a Petri plate containing *E. coli* colonies expressing EGFP was used. The 1PEF and 2PEF signals were collected, and the ratio of the 2PEF/1PEF was calculated for each colony on the Petri plate. The noise in the ratio of the two signals was assumed to be dominated by the noise from the laser amplification process. The signal to noise ratio was calculated by taking the average 2PEF/1PEF signal and dividing it by the standard deviation of the 2PEF/1PEF signal. The laser amplification limited signal to noise ratio for our 1 kHz laser system was found to be 9.

Finally, we will assume that for a 100 MHz repetition rate laser the signal to noise ratio is limited by the noise intrinsic to the detection system. The noise of the camera used in our system was measured by taking multiple images with no illumination on the camera and an 8 second exposure time. The standard deviation of the resulting pixel values was calculated and found to be 17.9 counts. Using this number the signal to noise ratio for the 2PEF image taken with a high repetition rate laser can be found using the following equation:

$$camera\ limited\ signal\ to\ noise\ ratio\ = \frac{2PEF_k}{17.9 * 10}$$

In this equation $2PEF_k$ is the fluorescence detected from one colony of bacteria in one vertical pass of the illuminating laser stripe. The factor of 10 multiplying the noise per camera pixel per image comes from the fact that for this camera each colony of bacteria is about 10 pixels across.

Supplementary Fig. 4a shows the estimated maximum number of samples that can be screened per day as a function of the average laser power based on the above assumptions and calculations. The red dashed lines correspond to an ultrafast oscillator laser with a 100 MHz pulse repetition rate and the black dashed lines correspond to a 1 kHz pulse repetition rate typical of a femtosecond regenerative amplifier. In both cases we assume that the pulse duration is 100 fs. The open red circle in Supplementary Fig. 4a shows the maximum screening rate using a generic 2PEF microscope setup utilizing a diffraction-limited focus spot and 10 mW average power, if we assume that the typical video-rate spatial beam scanning could be extrapolated from 100x100 $\mu m^2$ area to 9x9 $cm^2$ sample area. Note that increasing the average laser power (thin red line) from 10 mW to 100 mW would, in principle, be sufficient to meet the minimum average sample imaging rate (solid thick blue horizontal line). However, when arriving at this apparently favorable estimate we had to assume that all *E. coli* colonies grow such that they have identical spatial properties. In practice, the growth is always heterogeneous, and individual colonies end up having very different thicknesses, sizes and shapes. In addition, the concentration of FP expressed in each colony is different from one colony to another. To achieve the necessary quantitative reproducibility, the fluorescence signal from the FPs needs to be excited and collected uniformly throughout the whole thickness of each colony. As a result, the Rayleigh range of the excitation beam focus cannot be very short, and must be at least as large as the maximum thickness of the colonies, about 1 mm. Furthermore, for the

purpose of quantitative evaluation of the 2PEF efficiency, it is necessary to calibrate the 2PEF signal with respect to the 1PEF signal collected from the same colony, which also implies that the two-photon excitation photon flux must be uniform throughout the thickness of the sample. Our model calculation shows that under such realistic conditions the optimum spot diameter for the 100 MHz excitation is about 16 – 200 μm, depending on the laser power. The thick red dashed line in Supplementary Fig. 4a shows the minimum average power that accounts for the heterogeneity of colony growth. The result indicates that with the 100 MHz femtosecond oscillator about 1W of average power would be needed to meet, simultaneously, the minimum screening rate as well as the minimum S/N value.

A spot diameter of 16 μm means that about $10^7$ beam positions will be needed to cover the area of a standard Petri plate. This type of high precision, large area, focused beam scanning may become technically involved. An alternative would be a generic 1 kHz pulse rate regeneratively amplified femtosecond laser. This type of femtosecond amplifier typically operates at about $10^5$ times lower pulse repetition rate (1 kHz) than the femtosecond oscillators, while providing a factor of about $10^5$ higher peak photon flux. The higher peak intensity allows the laser spot diameter to be expanded by several orders of magnitude, up to several millimeters, while maintaining the required minimum S/N ratio. Supplementary Fig. 4b shows an example of how just about 20x20 partially overlapping beam positions can cover the entire sample. A smaller number of excitation beam positions is the key for achieving high accuracy and fast throughput. The fundamental drawback of this approach is that amplified lasers exhibit quite high, up to 10%, root mean square energy fluctuations, whereas in the 100 MHz oscillator pulses such fluctuations are minimal. To maintain the required minimum S/N ratio, the 2PEF signal obtained using an amplified femtosecond laser source requires additional averaging and therefore longer collection time. The upper limit of the shaded area in Supplementary Fig. 4a shows the minimum average laser power in the case of an ideal noise-free amplifier, and the lower limit shows the corresponding limit in the case of 10% root mean square energy fluctuations. Comparison of the shaded area to the red dashed line shows that even with a relatively high noise level the 1 kHz laser system offers a distinct advantage over the 100 MHz pulses.

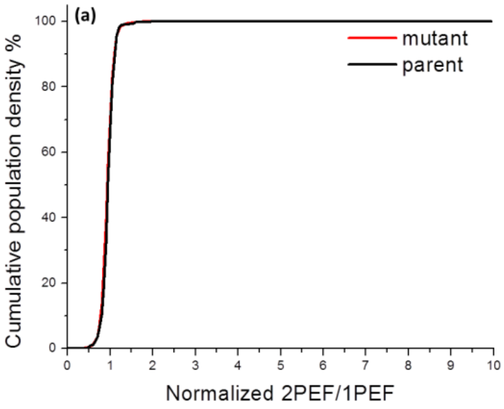

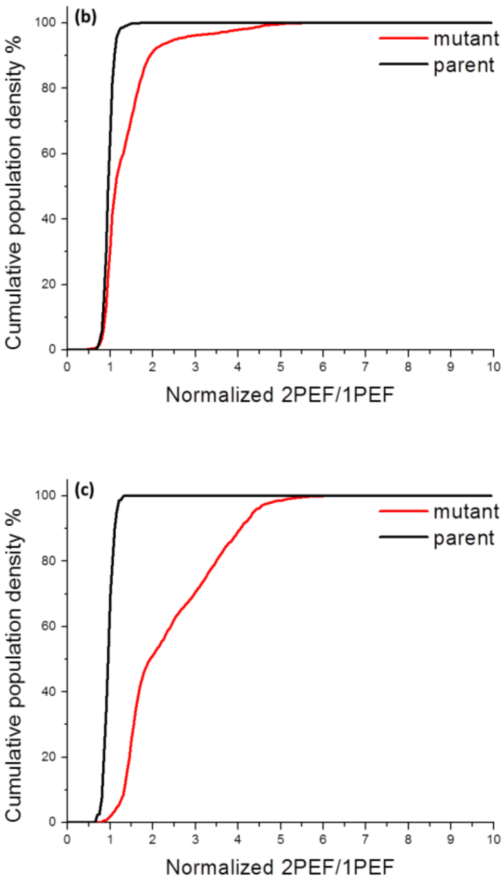


**Supplementary Figure 2| Cumulative distribution functions of fluorescence data of randomly mutagenized EGFP.** Cumulative percentage of the colonies (vertical axis) with a particular ratio value $\eta$ (horizontal axis). (a), The 1st generation library. (b), The 2nd generation library. (c), The 3rd generation mutagenized library.

**Plots of the effective two-photon cross section for selected mutants.**

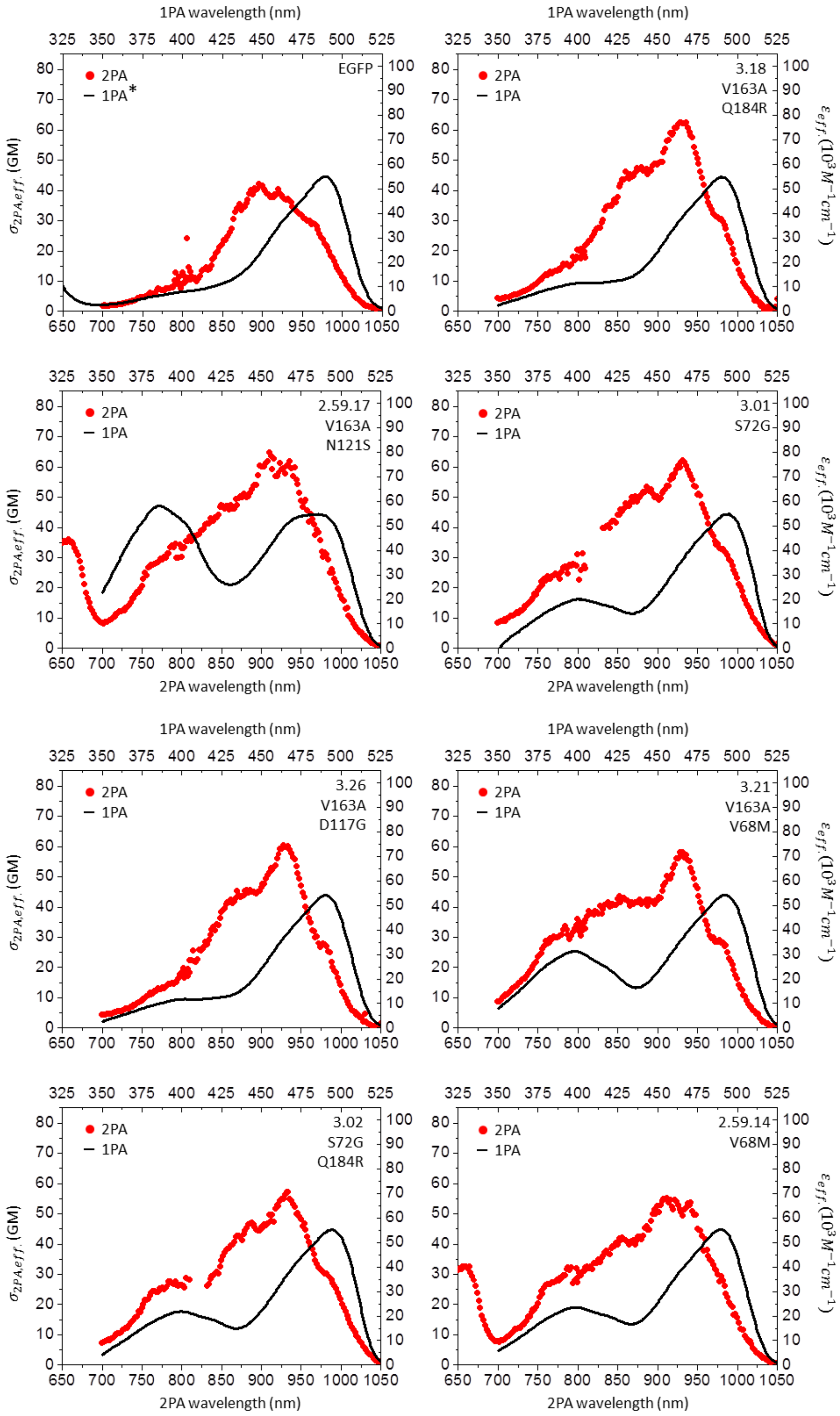

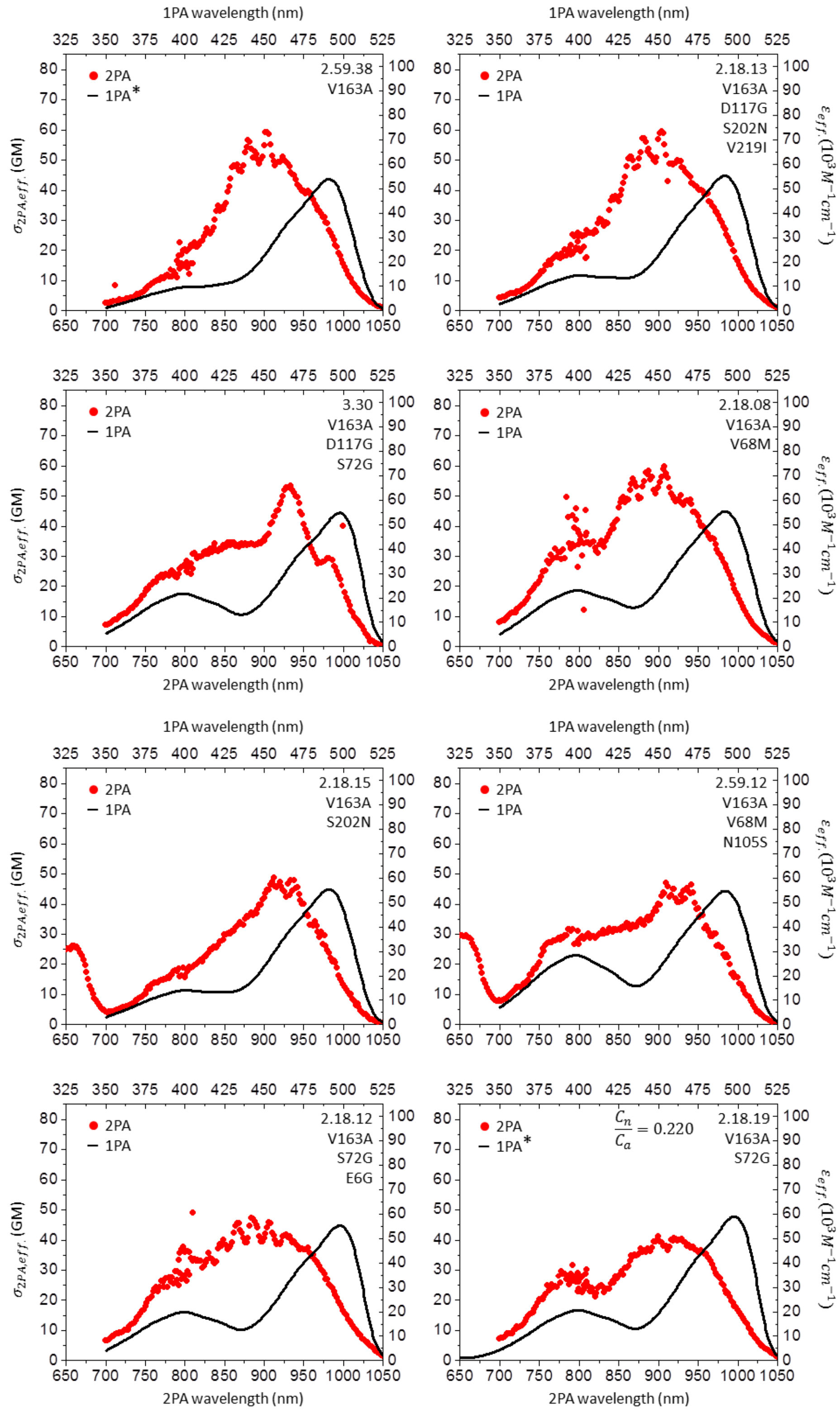
1PA wavelength (nm)
2PA wavelength (nm)
$\sigma_{2PA,eff.}$ (GM)
$\varepsilon_{eff.}(10^3 M^{-1} cm^{-1})$
2PA
1PA*
2.59.38
V163A
1PA
2.18.13
V163A
D117G
S202N
V219I
3.30
V163A
D117G
S72G
2.18.08
V163A
V68M
2.18.15
V163A
S202N
2.59.12
V163A
V68M
N105S
2.18.12
V163A
S72G
E6G
$\frac{C_n}{C_a} = 0.220$
2.18.19
V163A
S72G

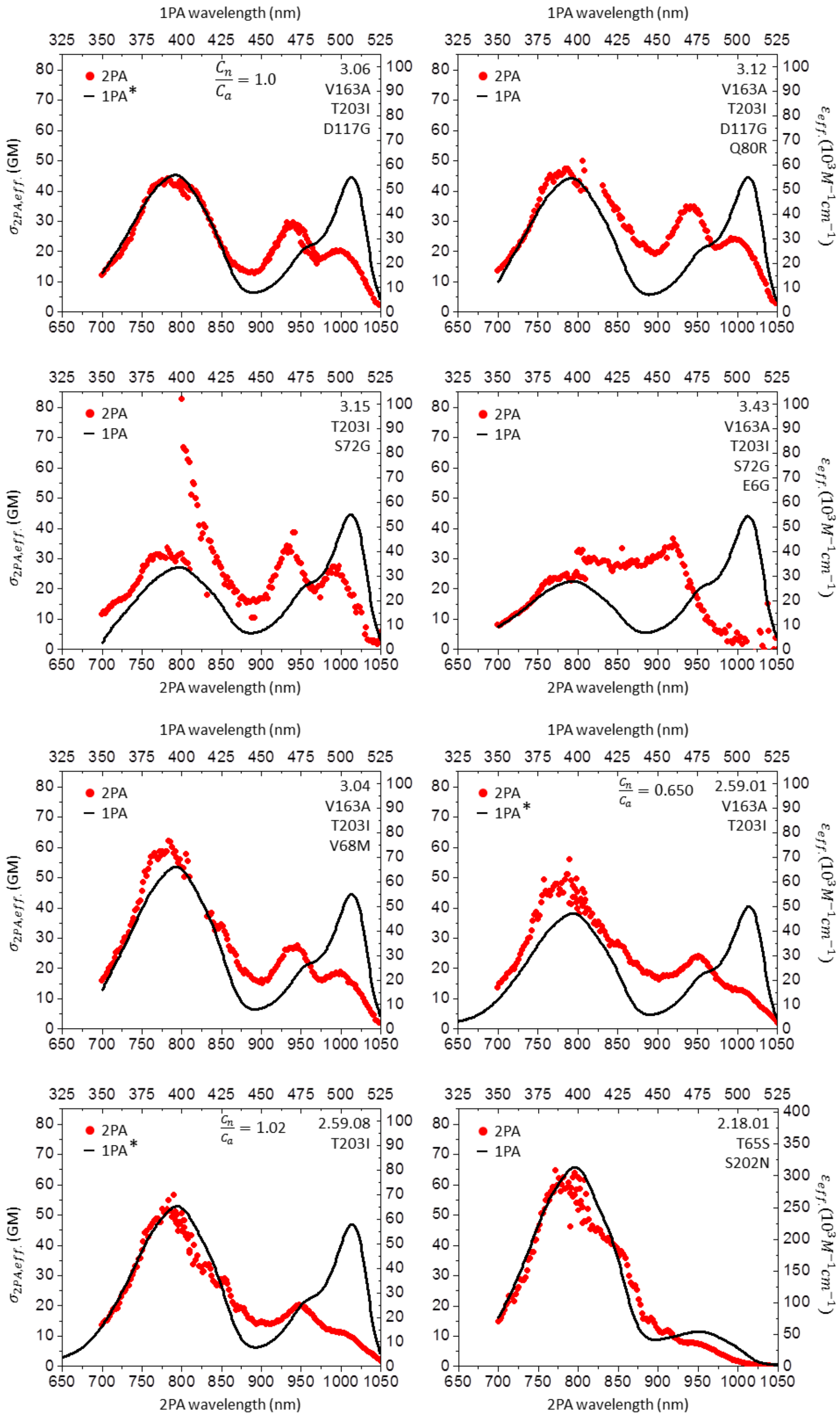


**Supplementary Figure 3| Absorption spectra.** Here all of the effective two-photon cross section spectra are shown. The effective extinction coefficients spectral shapes are overlaid on the plots for reference. The plots are arranged in the same order as in Table 1 in the main text. *For 1PA spectra with a star the absolute values of the extinction coefficients were explicitly measured using the method outlined in Supplementary

Information. For all other mutants the peak 1PA of the anionic form of the chromophore was assumed to be 55,000 $M^{-1}$ $cm^{-1}$. The ratio of the concentration of the two forms is shown for those mutants for whom it was measured. The numbers in the upper right hand corner of each plot designate different mutants.

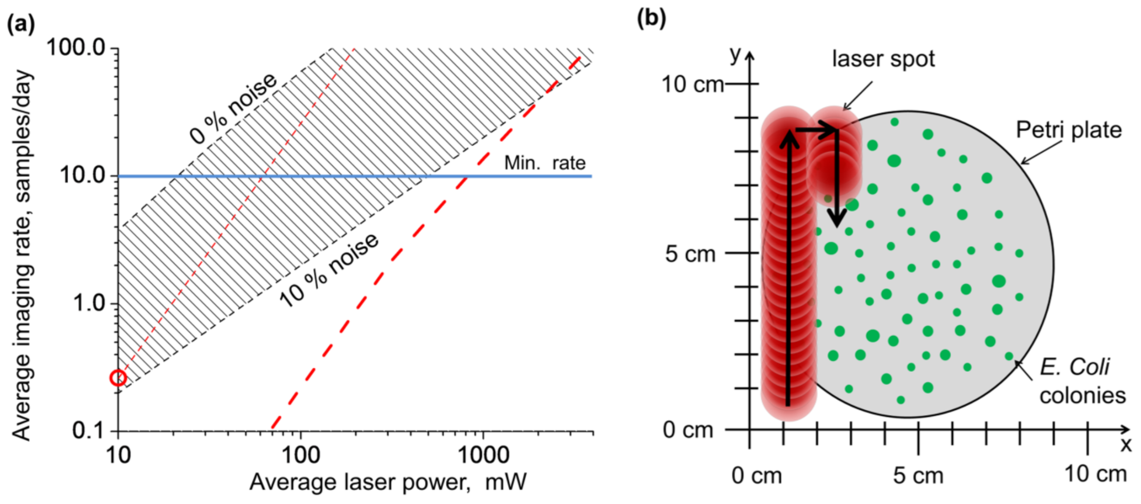


**Supplementary Figure 4| Imaging rate estimation versus laser parameters.** (a) Estimated average imaging rate (vertical axis) as a function of the average laser power (horizontal axis) assuming S/N=10. The thick blue solid line shows the required minimum imaging rate of 10 samples per day. The red circle represents a 100 MHz repetition rate laser using a 2PEF microscope setup with diffraction-limited focusing and video rate scanning. The thin red line represents an extrapolation to higher average powers. The red dashed line represents a 100 MHz laser, taking into account heterogeneous growth of *E.coli* colonies and the associated decrease of imaging rate due to an increase of the minimum laser spot size. The black shaded area represents a 1 kHz pulse rate amplified laser with root mean square energy fluctuations between 10% (lower dashed line) and 0% (upper dashed line) (b) Schematic representation of the spatial beam scanning with a 1 kHz laser beam (red circles). The shaded gray area is the Petri plate containing colonies of *E. coli* bacteria (green dots). The whole plate is covered by less than 400 beam positions.
